# Olfaction and topography, but not magnetic cues, control navigation in a pelagic seabird: displacements with shearwaters in the Mediterranean Sea

**DOI:** 10.1038/srep16486

**Published:** 2015-11-09

**Authors:** Enrica Pollonara, Paolo Luschi, Tim Guilford, Martin Wikelski, Francesco Bonadonna, Anna Gagliardo

**Affiliations:** 1 Department of Biology, University of Pisa, Via Volta 6, 56126 Pisa, Italy; 2 Department of Zoology, University of Oxford, South Parks Road, Oxford, UK; 3 Max Planck Institute for Ornithology, Department of Migration and Immuno-ecology, Schlossallee 2, Radolfzell 78315, Germany; 4 Dept. of Biology, University of Konstanz, 78468 Konstanz, Germany; 5 CNRS/CEFE, 1919, route de Mende, 34293 Montpellier Cedex 5, France

## Abstract

Pelagic seabirds wander the open oceans then return accurately to their habitual nest-sites. We investigated the effects of sensory manipulation on oceanic navigation in Scopoli’s shearwaters (*Calonectris diomedea*) breeding at Pianosa island (Italy), by displacing them 400 km from their colony and tracking them. A recent experiment on Atlantic shearwaters (Cory’s shearwater, *Calonectris borealis*) breeding in the Azores indicated a crucial role of olfaction over the open ocean, but left open the question of whether birds might navigate by topographical landmark cues when available. Our experiment was conducted in the Mediterranean sea, where the availability of topographical cues may provide an alternative navigational mechanism for homing. Magnetically disturbed shearwaters and control birds oriented homeward even when the coast was not visible and rapidly homed. Anosmic shearwaters oriented in a direction significantly different from the home direction when in open sea. After having approached a coastline their flight path changed from convoluted to homeward oriented, so that most of them eventually reached home. Beside confirming that magnetic cues appear unimportant for oceanic navigation by seabirds, our results support the crucial role of olfactory cues for birds’ navigation and reveal that anosmic shearwaters are able to home eventually by following coastal features.

Whilst the mechanisms of navigation in terrestrial environments by birds have been well studied, those of the potentially more challenging oceanic environment have been much less so. Broadly, homing pigeons, the best studied terrestrial birds, are now thought to navigate primarily using an olfactory map beyond their familiar area^1^,^2^,^3^ with an increasing dependence on visual landmarks as these become familiar^4^. Over the more featureless oceans, long distance navigation is much less well understood, but the involvement of olfactory cues is perhaps the best supported^5^,^6^, with several displacement experiments having failed to show a role for magnetic cues^6^,^7^,^8^,^9^,^10^,^11^,^12^.

Procellariformes have a particularly well-developed olfactory system and evidence has accumulated that these seabirds use olfaction in several behavioural tasks, such as to locate foraging areas with abundance of prey in the open ocean^13^,^14^, to find their nest among others in the dark^15^,^16^,^17^,^18^ and to recognise their mate^19^. One recent experiment^6^, in particular, has provided direct evidence of a navigational role of olfactory cues, showing that Cory’s shearwaters (*Calonectris borealis*)^20^ were able to home directly to their colony in the Azores after a 800 km displacement as long as they were not olfactorily impaired (whilst a magnetic disruption treatment caused no obvious decrement relative to controls). The authors concluded that the distribution of biogenic odours, reflecting plankton distribution in the ocean, might provide spatial information useful for a position finding mechanism in seabirds^5^,^8^,^9^,^11^. However, two anosmic shearwaters displaced from the Azores appeared able to orient more effectively once they had come within proximity of an island in the archipelago, suggesting that oceanic navigators might, like their terrestrial counterparts, utilise stable familiar landmarks once these come into sight.

Here, we aimed to explore further the potential role of visual landscape topography, magnetic, and olfactory cues in oceanic navigation using displacement experiments in an environment more richly bounded by coastal and island features where all cues may be available: the Mediterranean Sea. We studied Scopoli’s shearwaters (*Calonectris diomedea*), a close relative of Cory’s, which spend more than half of their annual cycle in the Mediterranean sea, breeding on small islands and along the coasts of several Mediterranean countries. After breeding they migrate into the Atlantic Ocean moving along the western African coast^21^. It seems likely therefore that breeding individuals will have considerable experience of large parts of the Mediterranean and its coasts, and we therefore predicted a shift from olfactory to landmark-based guidance. In a previous experiment with this species^7^, birds were apparently unaffected in homing to Linosa Island (Italy) by a magnetic disruption treatment, but this work was conducted before the advent of miniature on-board tracking technology so it is not possible to determine whether birds deprived of sensible magnetic cues had instead resorted to landscape features to home. Thus the question of magnetic navigation at this scale also remains unresolved.

Here, we released in the open sea, about 400 km from their colony, and tracked by GPS or Argos satellite telemetry, individual shearwaters either i) carrying magnets, ii) deprived of their sense of smell through irrigation of the olfactory mucosa with zinc-sulphate solution, or iii) subjected only to washing of the external nostril with sea water (controls), and analysed their navigational choices in relation to the environmental cues apparently available.

## Materials and Methods

### Birds

Thirty-two Scopoli’s shearwaters breeding at the colonies of La Scola rock (42.584° N; 10.106° E) and Punta Brigantina (42.568° N, 10.091° E) at Pianosa Island (Tuscan Archipelago, Italy) were used in this study, conducted during incubation in June 2012 and 2014 (see Supplementary Table S1 online for details). These colonies have been monitored for breeding success since a rat eradication programme on La Scola in 2001 when most accessible nesting birds were ringed (with some added during the current study). The number of nests in La Scola colony estimated before the present experiment ranged between 60 and 100 breeding pairs^22^. However at the time of the experiment the number of estimated nests was greater than one hundred. Subjects were selected for displacement within two nights of starting their incubation shift (which normally last 7–10 days between partner alternations^23^), to maximise motivation to return directly to continue their stint rather than forage. After capture birds were kept and transported in individual cardboard boxes (43 × 20 × 26 cm), with their egg left *in situ* at La Scola colony, but removed, replaced with a model, and incubated artificially for the birds at Punta Brigantina where rat predation remains a risk in unattended nests. This study was conducted under licences issued by ISPRA (permits n° T-A31 0018687 2014) and by the National Park of the Tuscan Archipelago (permit n° 0003066/2014). The experiment has been conducted according to the Italian regulation on animal welfare. The experimental procedures on the birds have been approved (permit n° 12498 03/10/2012) by the committee for the animal welfare of the University of Pisa (C.A.S.A.).

### Treatments

Before displacement birds were subjected to the same sensory manipulations as performed in^6^: anosmic (n = 14), magnetically disrupted (n = 9), and control (n = 9). Anosmia was achieved by washing the olfactory mucosa with 2.5 ml of 4% zinc-sulphate solution using a curved needle with rounded tip to each nostril^6^,^17^,^24^. Zinc-sulphate specifically affects olfactory cells, causing their necrosis^25^, with subsequent regeneration after several weeks. Control birds were subjected to external nostril washing with sea water, in order to subject them to a similar manipulation as the Anosmic birds, without touching the olfactory mucosa.

Magnetically treated birds had a semi-cylindrical PVC box (∅ 1 cm, length 3.5 cm) glued on the head, containing a strong cylindrical Neodymium magnet (∅ 5 mm, length 10 mm, total mass 3.9 g; magnetic moment 0.1 A.m^2^), which was free to tumble inside the box to produce an unpredictably variable artificial magnetic field stronger than ambient (as in^26^). The artificial field produced was about 60000 nT at 7 cm from the magnet, with the natural field being around 46000 nT in the study area.

### Tracking system

All Control and Magnetic birds, and 9 Anosmic birds (see Supplementary Table S1 online) were equipped with stripped IgotU GPS data loggers made water-proof with sealed heatshrink tubing. The switch of the IgotU logger was substituted by a magnetic switch to allow each device to be turned on at release. The disadvantage of this system is that the magnet on the bird’s head can accidentally interfere with the device when the head gets close to the switch. A brief approach results in the recording of an additional fix recognisable in the logged data, while a prolonged approach may result in a turning off of the device. Loggers were attached to the back feathers with water-proof marine tape (Tesa number 4651), and set to record position every 5 minutes. As it is known that some petrels species deprived of their sense of smell have difficulties in finding their burrows^15^,^16^,^17^,^24^, making tag recovery difficult, in 2012 experiments 5 more Anosmic birds were equipped with Argos-linked satellite transmitters (two models of 12 g PTTs- Microwave M-12G-S with solar panels, and Microwave PTT-100). These PTTs were light enough to be used on Scopoli’s shearwaters that are characterised by a smaller body size compared to the Atlantic Cory’s shearwater^20^. The PTTs were glued on a thin plexiglass rectangle (1 × 87 × 19 mm) which was fixed to the back feathers with marine tape.

### Releases

On the afternoon of the capture day, the cardboard boxes containing the birds were embarked on a boat travelling from Pianosa to Marina di Campo (Elba Island), then transported by car to Portoferraio (Elba Island) and embarked on a ferry to Piombino. The birds were then transported by car from Piombino to Livorno and in the evening boarded a ship travelling from Livorno to Barcelona (Spain), from where they were released in the Lion Gulf, about 400 km West of the colony and at least 100 km from the closest coastline. Three successive displacements have been performed (see Supplementary Table S1 online) and therefore the birds were not released at exactly the same point because of differences in the route followed by the ferry each time. To account for this, the mean home direction for each group was determined (see Supplementary Table S1 online for details). Releases began about 17 hours after departure from Pianosa island, with birds released astern at about 5 min intervals, rotating individuals from the treatments. During releases the coastline was not visible to the experimenter. Nests were monitored daily for recoveries over at least the next 8 days, and at least twice a week over the following month. Sometimes birds were not recaptured immediately after arrival back at the colony, and performed spontaneous foraging movements which we also recorded within 15 days after their displacement. The t test has been applied to compare the length and the maximal distance reached from the colony during the first trip between the smelling (C and M) and anosmic (A) shearwaters.

All original track data are available at the data archive Movebank (www.movebank.org, study name “Pianosa shearwaters”, DOI 10.5441/001/1.tc76g560).

### Data analysis

Homing route reconstruction was made with all the GPS fixes obtained, but in the successive analyses we only considered fixes for which calculated speed was higher than 10 km/h, to eliminate periods likely to have been resting on the sea surface or foraging^27^. Improbable Argos locations were filtered out using a maximum speed threshold of 80 km/h between successive locations. Since the duty cycle of the PTTs was set 10 h ON and 48 h OFF, Argos locations were only used to assess homing success and the distance from home over time (every 24 hours after release); in case the actual PTT location was not available an interpolation between two successive locations was made. Homing success amongst groups was compared by applying the χ^2^-goodness of fit to a 2 × 3 contingency table reporting the number of birds homed and lost in the three treatments (rows: homed, lost; columns: C, A, M). Homing performance was also evaluated from the propensity of birds to approach the colony over the first five days following release. Between group differences were tested using Two Way RM ANOVA and the Student-Newman-Keuls test for multiple comparisons. Initial oceanic orientation was evaluated by analysing the GPS track over the first 60 km radius from the release point (where a view of land was unlikely), and calculating a mean vector of the directions taken by the bird from each recorded fix to the next. Each mean vector is therefore representative of the flight path of a shearwater in the open sea, likely in absence of visual topographical cues. The same analysis was conducted for track sections ranging from the release point to the first fix located near the coast (distance less than 20 km), or up to the coast.

The mean vector distributions were tested for randomness with the One Sample Hotelling test. We performed One Way ANOVA or a Kruskall-Wallis test (for non normal distribution) applied to the mean vector length to compare the tortuosity of the initial flight path, and to the deviation of the mean vector directions from homeward to evaluate differences in orientation amongst the three experimental groups. Multiple comparisons were performed with Bonferroni or Dunn’s tests.

For analysis on the whole tracks we considered the flight path from the release point up to 10 km from the colony (since birds often reached waters local to Pianosa long before they made landfall at night). Flight path efficiency indices (EI) were obtained by dividing the beeline between the first and the last considered fix by track length. In the case of interrupted tracks, the distance from the last recorded fix to 10 km from home was added to the flight path length for computing the EI. EIs were compared amongst treatments with One Way ANOVA, and Bonferroni t tests for multiple comparisons.

To assess the preference of birds for travelling near the coast rather than over open sea we considered the percentage of fixes that were less than 40 km distant from the closest coastline. This analysis was conducted from the section of each track ranging from the release point up to a Longitude equal to 9.3407° E (corresponding to Capo Grosso, the northernmost part of Corsica Island, France) in order to exclude the Tyrrhenian sea where, because of the presence of many small and big islands, it is almost impossible not to be within 40 km of land. For this analysis we calculated the number of fixes distant less than 40 km from the closest coastline using a spatial analysis procedures with QuantumGIS (http://www.qgis.org/), allocating birds to “near” or “far” categories, according to whether more or fewer than 50% of their fixes were closer than 40 km to the coast. We applied the χ^2^-goodness of fit test on the number of birds of the three treatment groups falling in the two categories.

To test the hypothesis that anosmic birds were able to home exploiting visual topographical cues we estimated the decision point at which the birds started to move efficiently towards home using a backward path analysis^12^,^28^. Starting from home and moving backward along each bird’s path we plotted the backward beeline distance from home of each track point against the bird path length from home to the current fix; then, we determined the point (decision point) at which the distance from home stops increasing linearly with respect to the backward path length. If this point was homeward of the Longitude line 9.3407 East (see above), the decision point immediately before was considered in the analysis. In case of ambiguity we chose the decision point less favourable to our hypothesis. Finally, we compared with the χ^2^-goodness of fit test the number of birds in each group that had approached the coastline (at a distance less than 10 km) before the decision point.

## Results

All displaced Control (n = 9) and Magnetic (n = 9) treatment birds returned to the colony. Two Magnetic birds had lost their magnets by recapture, one of which had the track interrupted (see below). However, additional data recorded by the loggers (unexpected triggers of off/on switches) indicated that the magnet briefly got close to the GPS switch (see Materials and methods), revealing that the magnet was on the head of these two birds at least after entering Tyrrhenian sea East of Corsica Is. Three GPS-equipped Anosmic shearwaters were never found at the colony during the monitoring period and their egg failed. No difference in the homing success between the three groups emerged (number of homed vs non homed birds, χ^2^ goodness of fit, p > 0.1).

**Figure 1:**
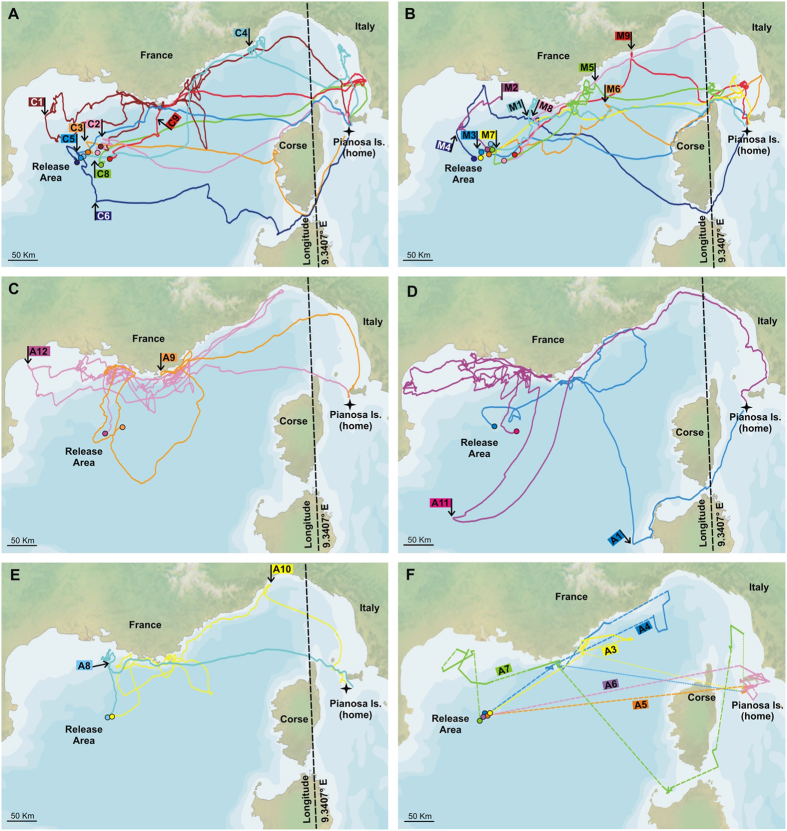
Homing paths of the shearwaters over a Natural Earth map ( http://www.naturalearthdata.com). In the panels reporting the GPS tracks the longitude delimiting the section of the tracks analysed for the propensity of the birds to fly near or far from the coast (see methods for details). For each track the circle represents the release point; when present, the arrow at the basis of the flag reporting the individual code represents the decision point (see text for details). Panel (**A**) GPS tracks of Control birds. Panel (**B**) GPS tracks of Magnetic birds. The flag of M2 is placed at the end of the interrupted track; the decision point has not been determined because this track is too short. Panel (**C**–**E**) GPS tracks of Anosmic birds. Panel (**F**) PTT tracks of Anosmic birds. Broken lines represent the beeline between the last fix of a recording session and the first fix of the following recording session. Dotted lines represent the beeline between the last fix recorded and home.

We obtained 8 complete Control tracks (one bird lost its GPS) (Fig. 1A), and 6 complete and 3 interrupted Magnetic tracks (Fig. 1B), one of which was short (about 80 km) so that it was included only in the initial orientation analysis. The interruption of the Magnetic tracks was caused by the head magnets inadvertently getting sufficiently close to the GPS device to trigger the on/off switch (see Material and methods). From Anosmic birds we obtained 6 GPS tracks suitable for all the analyses (Fig. 1C–E) and 5 PTT tracks (Fig. 1F), that due to the low number of localizations could be used only in the homing performance analysis.

**Figure 2:**
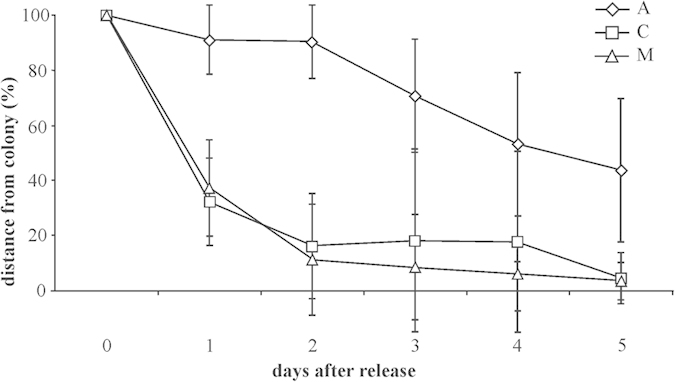
Mean distance from the colony, expressed as percentage of the distance at the release site, of the three groups of shearwaters (C, controls n = 8; M, birds bearing mobile magnets, n = 8; A, anosmic birds n = 11) from the time of release (indicated with 0) up to day 5 after release. The bars represent the 95% confidence limits.

Both Control and Magnetic subjects approached their home island faster than Anosmic birds over the five days following release (Fig. 2; Two-way RM ANOVA p < 0.001; Bonferroni test p < 0.001 Anosmic vs. Magnetic and Anosmic vs. Controls; p > 0.5 Control vs. Magnetic).

**Figure 3:**
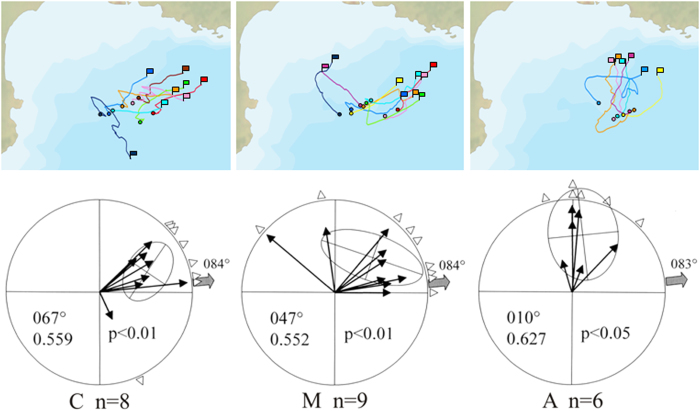
Sections of the tracks (colours as in Figure 1) from the release point (circle) up to 60 km away from it (flag) and the relative diagrams reporting their orientation: C, control birds, n = 8; M, birds bearing magnets n = 9; A, anosmic birds, n = 6. Each mean vector indicates the mean orientation of a bird’s track. The mean vector distributions are tested for randomness with the Hotelling test (95% confidence ellipses are reported). Triangles at the periphery of the circles represent the tracks’ mean directions. The grey arrow indicated the mean home direction for each experimental group. Second order mean vector length and direction are reported.

Figure 3 shows the initial portion of the GPS tracks when birds were in open sea (<60 km from the release site) and the relative mean vector distributions. In general, the initial orientation of the birds within each group was quite consistent. Evident outliers were one C bird and two M birds initially heading S and NW, respectively. The C bird and one of the M-birds (M1) changed their route soon after this first segment, eventually heading towards home (Fig. 1A,B), while for the second M-bird (M2) we only recorded the initial part of the route. Mean vector distributions were all different from random (Hotelling test; C, n = 8, second order mean vector r = 0.55 α = 067° p < 0.01; M, n = 9, r = 0.55 α = 047° p < 0.01; A, n = 6, r = 0.63 α = 010° p < 0.05). However, for the Anosmic birds the 95% confidence limits do not include the mean home direction (A, mean home direction 083°, 95% c.l. 330°−059°) while for both Control and Magnetic groups homeward was either included in the 95% confidence limits (C, 084°, 95% c.l. 043°–105°) or coincident with one of them (M, 084°, 95% c.l. 344°−084°). The three experimental groups displayed a difference in orientation with respect to the home direction (Kruskall-Wallis test applied on the angular distance between each vector and the home direction, p < 0.05; median angular distance Control 30°, Magnetic 28°, Anosmic 80°). In particular, the Anosmic group exhibited greater deviation from homeward than the Control group (Dunn’s test C vs A p < 0.05) while no significant difference emerged in the other comparisons (C vs M p > 0.5 and A vs M p > 0.05).

The same analysis performed on the track sections ranging from release to the first fix closer than 20 km to the coast produced very similar results: the three mean vectors distribution were different from random, but only Control and Magnetic treatment birds were significantly homeward oriented (Hotelling test; C, n = 8, second order mean vector r = 0.67 α = 069° p < 0.001, 95% c.l. 043°–097°; M, n = 8, r = 0.67 α = 052° p < 0.01, 95% c.l. 019°–085°; A, n = 6, r = 0.63 α = 018° p < 0.01, 95% c.l. 356°−043°). Also considering the section of the tracks from the release point up to the coast the three experimental groups displayed a difference in orientation with respect to the home direction (One Way ANOVA applied on the angular distance between each vector and the home direction, p < 0.005; mean angular distance Control 23°, Magnetic 36°, Anosmic 65°). In this analysis the Anosmic group exhibited a greater deviation from home than both the Control and the Magnetic group (Bonferroni test C vs A p < 0.005; M vs A p < 0.05), while no significant difference emerged between C and M (p > 0.5).

**Figure 4:**
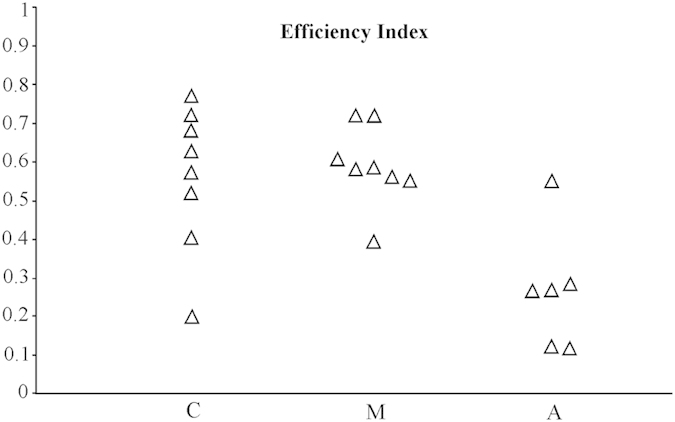
Each triangle represents the efficiency index of a GPS track. C, control birds n = 8; M, birds bearing magnets n = 8; A, anosmic birds, n = 6.

Whole track efficiency indexes (calculated only on GPS tracks; see Material and methods) differed significantly across the three groups (One Way ANOVA, p < 0.005; C n = 8 mean EI = 0.559±0.187; M n = 8 mean EI = 0.588±0.103; A n = 6 mean EI = 0.266±0.158; see also Fig. 4). In particular, Anosmic birds displayed significantly lower EI than the others (Bonferroni, C vs A p < 0.01; M vs A p < 0.005; C vs M p = 1), while no difference occurred between the control birds and magnetic treatments.

**Figure 5:**
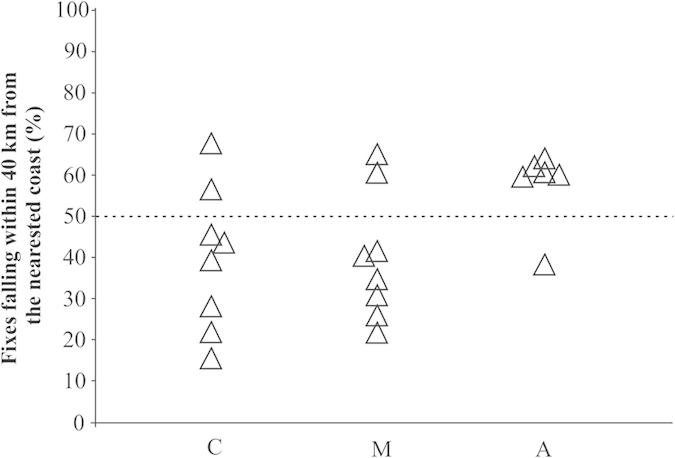
Each triangle represents the percentage of fixes of each track falling within 40 km from the nearest coast. C, control birds n = 8; M, birds bearing magnets n = 8; A, anosmic birds, n = 6. The birds with a percentage greater than 50% were assigned to the category “near”, the other ones to the category “far” (see text).

Considering the section of the tracks from the release point up to the longitude of Corsica, we observed a difference in the flight paths with respect to coastlines across the three experimental groups (χ^2^ goodness of fit performed on the number of birds assigned to the categories “near” and “far”, see Material and methods, χ^2^ = 6.14, p < 0.05, see Fig. 5). In fact, most of Anosmic birds (5 out of 6) preferred to fly along the coast, in contrast to the other two groups (both C vs A and M vs A χ^2^ = 4.67 p < 0.05; C vs M χ^2^ = 0 ns).

The decision points (the point at which the distance from home stops increasing linearly with respect to the backward path length) determined with the backward analysis of the tracks (see Material and Methods) are marked in Fig. 1A–E. The number of birds that presumably viewed the coast before their decision point was significantly different in the three groups (χ^2^ = 7.88, p < 0.025). While all the anosmic birds (n = 6) had approached the coast before the decision point (A vs C χ^2^ = 7.87, p < 0.01; A vs M χ^2^ = 4.2, p < 0.05), only 2 out of 8 control birds and half magnetic birds (n = 8) did so (C vs M χ^2^ = 1.07, p > 0.2).

With respect to the impact of the experimental treatments on the reproductive success of the birds involved in the experiment, we observed that four out of eleven homed Anosmic birds raised their chick successfully. Since seven out of nine homed C, and six out of nine homed M raised their chicks (χ^2^ = 3.848 p > 0.1), the reproductive success of the A birds that had homed was not significantly lower than the other two groups. Even comparing the anosmic and the olfactorily competent (C and M together) birds’ reproductive success, no difference emerged (χ^2^ = 3.619, p > 0.05). This suggests that no side effect of zinc-sulphate treatment on the birds’ biology occurred.

The total reproductive success of pairs involved in the experiment was compared to that of other accessible nests in the colony (85 nests checked over the two years). The reproductive success of the displaced birds was significantly lower than that of the non displaced shearwaters (χ^2^ = 4.163, p < 0.05). On the whole we estimated that the impact of our experiment on breeding success resulted in 8 chicks fewer produced per year of the experiment. However, we are confident that the dynamics of the colony was not negatively impacted in the long term.

The GPS loggers recorded the spontaneous movements of 3 C, 1 M and 3 A shearwaters that were recaptured in the subsequent days after their homing back to the colony (see Supplementary Fig. S1 and Table S2 online). Daily movements were tracked for all the birds in a variable number depending on the number of days between the homing and the recapture (mostly 1–3; for one A bird 10 spontaneous flights were recorded). Exception made for one A bird (see below) all the spontaneous flights recorded lasted less than 24 hours (about 19 hours on average) No difference between the olfactorily unimpaired (C and M, n = 4) and anosmic birds (A, n = 3) was found in the first trip length (t test p > 0.2; unimpaired birds mean 97±35 km; anosmic birds 146±60 km) and in the maximal distance reached (t test p > 0.2; unimpaired birds 21±12 km; anosmic birds 34±10 km). In addition, for one A bird (#TH0433) we documented a four days long trip up to the northern tip of Corsica. This area is within the habitual foraging ground of the birds of Pianosa colonies^29^. This trip was performed after 9 days from its return to relieve its partner at the nest, that is when its motivation to forage is likely high^23^.

## Discussion

The initial orientation of shearwaters displaced some 400 km from their colony, and released out of direct sight of land, is approximately towards the colony in both control and magnetically disrupted birds. Consistently, the whole routes of these two groups were clearly directed towards home (Fig. 1 A,B), and indeed these birds covered more than the 80% of the distance from the release point to the colony in only two days (Fig. 2). This suggest that both groups of birds are capable of establishing their approximate location relative to home at this distance, and that information from the earth’s magnetic field is not necessary for doing so. Consistently with previous work^6^,^30^ both groups are also capable of taking relatively straight oceanic paths towards home, indicating that a detailed view of land features is also not necessary for homing on this scale. Anosmic birds by contrast show significant orientation on release, but towards the north and not towards the colony, despite being magnetically intact. This supports the view that olfactory information is crucial for determining location over the open ocean^6^ even within the bounded environment of the Mediterranean Sea. In accordance with this view, control and magnetically disrupted birds return to their colony equally quickly, whilst anosmic birds take significantly longer and, both from visual inspection and efficiency index analysis, show much more tortuous routes, supporting a role for olfaction in true navigation. The lack of navigational effect of magnetic disturbances is in line with the outcomes previous experiments in other Procellariformes species^6^,^7^,^8^,^9^,^10^,^12^,^31^.

Nevertheless, the eventual homing success of anosmic birds is not different from controls or those with disrupted magnetic perception, with all but three anosmic subjects returning before the end of the observation period, suggesting that there is an alternative source of information available for homeward guidance. Visual landmark cues were not manipulated directly, but an analysis of the points at which efficient homeward movement was initiated indicates that whilst controls and magnetically disturbed birds often started homeward far from land, anosmic birds had invariably approached a coastline first. Furthermore, the tracks of anosmic birds were far more likely to be along coastlines than were those of birds able to smell. This strongly suggests that homing was achieved by anosmic birds either by following coastlines as a form of search strategy, or by recognition of land features previously encountered within the Mediterranean (or by a combination of both). The fact that birds do not always orient in the homeward direction on approaching a coast, some showing striking reversals, and sometimes fail to take clear shortcuts towards the colony instead following the coastline circuitously, lends some support to the idea that any map of land features based on familiarity must be relatively sparse or local.

Although experiments in homing pigeons have excluded side effects of zinc sulphate treatment^32^,^33^, one may argue that it might have side effects on motivation to home in wild birds. This possibility has been recently excluded by an experiment on the closely related Cory’s shearwaters^17^. Two groups of shearwaters (zinc sulphate-treated and sham treated with saline solution) were displaced at night at short distance from their breeding colony in Selvagem Island, Portugal, where these birds are known to come back to the colony either during the day or the night. Both groups showed an high motivation to home but, while the control birds homed in a few hours during the night, the anosmic shearwaters were able to find their own nest among the others only at dawn, exploiting visual cues. These findings clearly show that the only effect of the treatment was an olfactory impairment that prevented anosmic shearwaters to efficiently locate their nest at night.It is known that seabirds are attracted by odours that are potential indicators of the presence of food and that they are likely to use odours to identify prey abundant areas in open ocean far from the coast, although olfaction is not strictly needed for prey detection and hunting^13^,^14^,^34^. It might therefore be argued that anosmic shearwaters took longer to home simply because they had more difficulty finding food sources rather than because of navigational impairment. However, for all birds the motivational state at the release site was very likely to be for homing rather than foraging, as they were captured and displaced on the same day or one day after their return from a foraging trip, when they would have been expecting to fast for up to ten days whilst on incubation duty (see Supplementary Table S1 online). Indeed, both C and M groups initially did not show any major sign of a motivation to forage, being clearly homeward oriented upon release and then returning to their colony with quick, direct routes. The foraging grounds of Pianosa shearwaters are all located near conspicuous topographical features (Corsica island, Ligurian sea, Elba island, Montecristo island), that can be located also without the aid of the smell. If the anosmic birds were not impaired in navigation, but only in finding food in the open sea, they could have oriented eastward and stopped in their habitual foraging grounds locating them thanks to both visual topographical cues and activity of other seabirds, as they did after their return to the colony. Their homing time would have been comparable to that of C and M, some of which stopped on their way back for feeding in their habitual foraging areas. By consequence, the fact that soon after release the A birds displayed an initial orientation significantly different from the home direction (see Fig. 3) and homed with longer, more tortuous routes, can be reasonably explained with a specific navigational impairment, rather than with a lower efficiency in finding food. In fact, the reproductive success of the homed anosmic birds did not significantly differ from that of the olfactorily unimpaired shearwaters, indicating that they were efficient in providing food for the chicks. In addition, once they reached the colony the homed A birds seemed to perform spontaneous movements back and forth to the colony efficiently within their familiar area^29^. This supports the hypothesis that for shearwaters olfaction is needed for navigation in unfamiliar and/or visually featureless environments, while visual cues provide auxiliary navigational information within familiar areas, as reported for homing pigeons^2^,^33^.

Most of the anosmic shearwaters headed north soon after release in open sea. As this is the direction of the nearest coast, this raises the possibility that the birds might have seen the presence of land in that direction and therefore approached the coast in order to inspect topographical features potentially useful for navigation. However, since the coastline was not in sight of the experimenter standing high in the ship, it is unlikely that a bird flying near the sea surface could have spotted the land at least from the same distance. It may not be excluded however that released birds came in visual contact with the coast when getting closer to it some time after release. Another possibility is that the anosmic birds, having no clue about their position with respect to home display a non-sense orientation in a preferred direction (PCD)^35^,^36^. The latter explanation is supported by the relative consistency of the directional choices of the Anosmic birds, that all ended up heading Northward, while in both Control and Magnetic groups one and two birds, respectively, displayed an orientation different from the rest of the birds belonging to the same experimental group.

In conclusion, our data confirmed the crucial role of olfactory cues in seabird navigation^6^, provided further evidence that magnetic disturbance does not affect navigational abilities of Procellariformes, and suggest that visual landmarks may constitute an alternative source of information allowing navigation in olfactory-deprived birds.

## Additional Information

**How to cite this article**: Pollonara, E. *et al.* Olfaction and topography, but not magnetic cues, control navigation in a pelagic seabird: displacements with shearwaters in the Mediterranean Sea. *Sci. Rep.* **5**, 16486; doi: 10.1038/srep16486 (2015).

## Supplementary Material

Supplementary Information
