# Supplementary material for: Olfaction and topography, but not magnetic cues, control navigation in a pelagic seabird: displacements with shearwaters in the Mediterranean Sea
Source: Sci Rep. 2015 Nov 9;5:16486. doi: 10.1038/srep16486 (PMC4637929; doi:10.1038/srep16486)
Supplement: Supplementary Information [file srep16486-s1.pdf]

Olfaction and topography, but not magnetic cues, control navigation in a pelagic seabird: displacements with shearwaters in the Mediterranean Sea

Enrica Pollonara, Paolo Luschi, Tim Guilford, Martin Wikelski, Francesco Bonadonna, Anna Gagliardo

Supplementary material

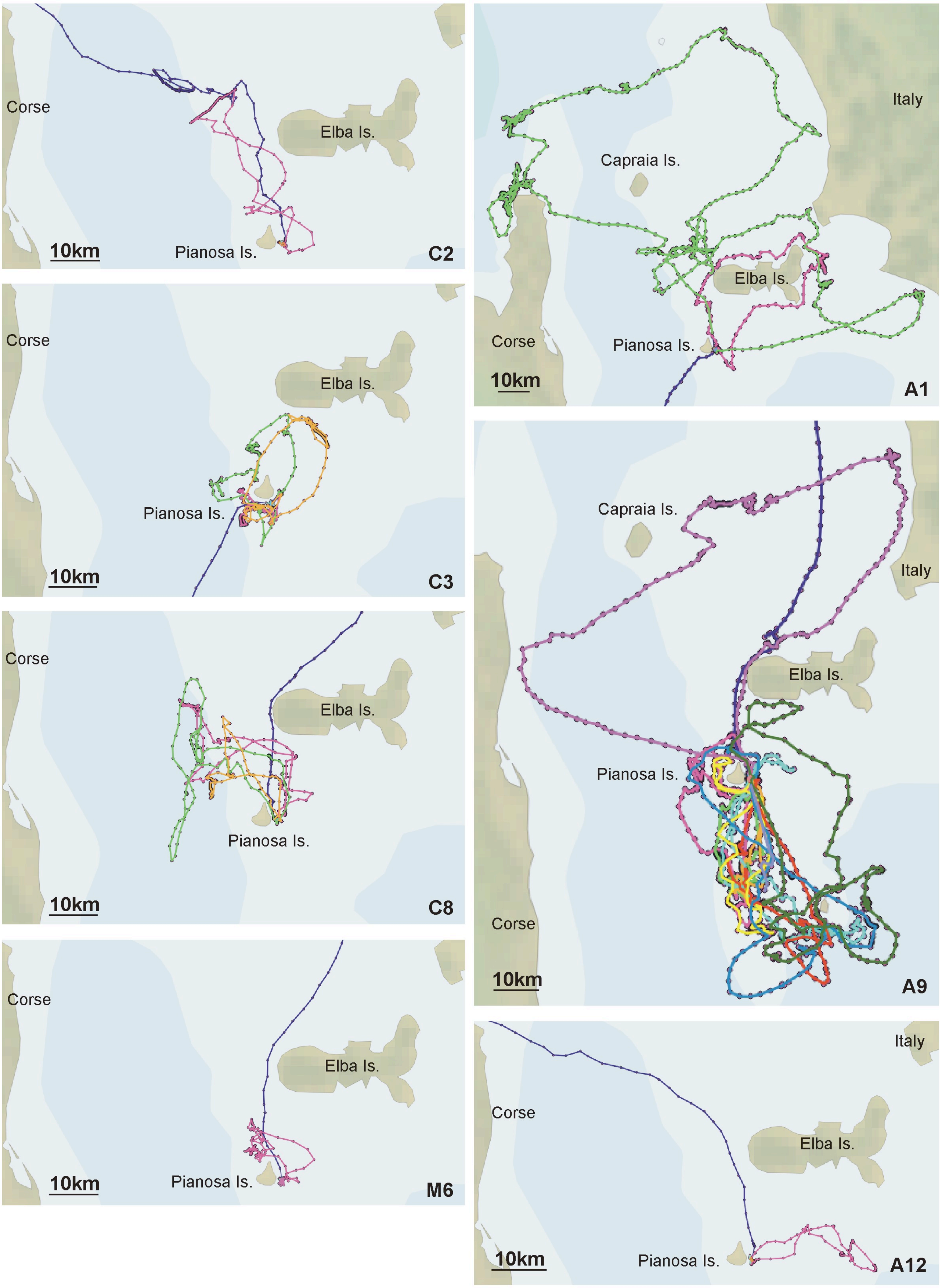

Figure S1. Spontaneous flights (see Supplementary Table S2 for the identification colour code), and the last part of the homing flight after displacement (dark blue line) are reported in the figure. The paths of the shearwaters are represented over a Natural Earth map (http://www.naturalearthdata.com). See main text and Supplementary Table S2 for other explanations.

Table S1

Tr	Track	Ring	Colony	SD	Release date/time	Release location	Tag	n	MV length dir°	Home dist dir°	Homing	Track analysis
C	C1	TJ3787	S	0	21/06/2012 11:41	5.034929 E 42.342415 N	GPS	28	0.668 60°	416 86°	26/06/2012 h	c[1,2,3,4,5]
	C2	TJ3757	S	0	21/06/2012 11:54	4.932396 E 42.308525 N	GPS	53	0.473 80°	425 85°	22/06/2012 h	c[1,2,3,4,5]
	C3	TJ9179	B	0	21/06/2012 12:15	4.803005 E 42.265091 N	GPS	43	0.561 72°	435 85°	22/06/2012 h	c[1,2,3,4,5]
	C4	TH1324	S	0	21/06/2012 12:38	4.62296 E 42.211235 N	GPS	23	0.947 84°	451 84°	25/06/2012 h	c[1,2,3,4,5]
	C5	TJ3758	S	1	21/06/2012 12:44	4.574549 E 42.202847 N	GPS	29	0.788 46°	455 84°	22/06/2012 h	c[1,2,3,4,5]
	C6	TH0429	S	1	21/06/2012 13:00	4.474911 E 42.171799 N	GPS	68	0.320 155°	464 84°	23/06/2012 h	c[1,2,3,4,5]
	C7	TA2200	S	0	26/06/2012 10:38	4.816201 E 42.239261 N	GPS			435 84°	28/07/2012 r	n
	C8	TK4887	S	1	22/06/2014 11:50	5.093163 E 42.134893 N	GPS	41	0.534 48°	414 82°	24/06/2014 h	c[1,2,3,4,5]
	C9	TK4890	S	0	22/06/2014 11:18	5.304507 E 42.197574 N	GPS	30	0.658 50°	396 83°	24/06/2014 h	c[1,2,3,4,5]
M	M1	TJ9189	S	0	21/06/2012 11:51	4.987313 E 42.317825 N	GPS	39	0.562 74°	420 86°	22/06/2012 h	c[1,2,3,4,5]
	M2	TA2195	S	0	21/06/2012 12:11	4.834994 E 42.281216 N	GPS	28	0.949 310°	433 85°	26/06/2012 r	i[2]
	M3	TA2249	S	1	21/06/2012 12:26	4.702586 E 42.241848 N	GPS	37	0.613 81°	444 85°	22/06/2012 h	c[1,2,3,4,5]
	M4	TA2157	S	0	21/06/2012 12:53	4.520641 E 42.180363 N	GPS	37	0.707 352°	460 84°	22/06/2012 h	c[1,2,3,4,5]
	M5	TC8805	S	1	26/06/2012 10:20	4.947409 E 42.285652 N	GPS	30	0.598 90°	424 85°	06/07/2012 r	i[1,2,3,4,5]
	M6	TH0410	S	0	26/06/2012 10:28	4.874951 E 42.259758 N	GPS	24	0.778 78°	430 85°	27/06/2012 h	c[1,2,3,4,5]
	M7	TE9218	S	1	26/06/2012 10:54	4.716255 E 42.197392 N	GPS	20	0.980 57°	444 84°	27/06/2012 h	c[1,2,3,4,5]
	M8	TK4891	S	0	22/06/2014 11:39	5.206323 E 42.168231 N	GPS	24	0.715 50°	404 83°	25/06/2014 r	i[1,2,3,4,5]
	M9	TK4889	S	0	22/06/2014 11:02	5.408251 E 42.227585 N	GPS	29	0.897 40°	387 84°	24/06/2014 h	c[1,2,3,4,5]
A	A1	TH0433	S	0	21/06/2012 12:04	4.896313 E 42.299709 N	GPS	64	0.301 21°	428 85°	27/06/2012 h	c[1,2,3,4,5]
	A2	TH0435	B	0	21/06/2012 12:19	4.745000 E 42.257000 N	GPS			439 85°	lost	n
	A3	TA2242	S	1	26/06/2012 10:22	4.915003 E 42.277896 N	PTT			427 85°	06/07/2012 r	lo[1]
	A4	TJ9188	S	0	26/06/2012 10:33	4.844896 E 42.250435 N	PTT			433 85°	28/07/2012 r	lo[1]
	A5	TJ3777	S	0	26/06/2012 10:43	4.78926 E 42.22625 N	PTT			438 84°	28/07/2012 r	lo[1]
	A6	TA2240	S	1	26/06/2012 10:48	4.746877 E 42.212343 N	PTT			441 84°	21/07/2012 r	lo[1]
	A7	TA2179	S	1	26/06/2012 10:59	4.675004 E 42.185043 N	PTT			447 84°	11/07/2012 r	lo[1]
	A8	TC8831	S	0	22/06/2014 11:38	5.173489 E 42.158507 N	GPS	25	0.891 5°	407 83°	25/06/2014 h	c[1,2,3,4,5]
	A9	TH0438	S	0	22/06/2014 11:10	5.385069 E 42.221226 N	GPS	71	0.289 344°	389 83°	25/06/2014 h	c[1,2,3,4,5]
	A10	TH0423	S	0	22/06/2014 11:22	5.273108 E 42.188067 N	GPS	36	0.781 44°	399 83°	26/06/2014 h	c[1,2,3,4,5]
	A11	TJ3795	S	1	22/06/2014 11:17	5.345146 E 42.209542 N	GPS	29	0.774 360°	392 83°	02/07/2014 h	c[1,2,3,4,5]
	A12	TA2247	S	0	22/06/2014 11:43	5.135368 E 42.147189 N	GPS	23	0.929 360°	410 83°	05/07/2014 h	c[1,2,3,4,5]
	A13	TA2248	S	0	22/06/2014 11:27	5.237152 E 42.177399 N	GPS			402 83°	lost	n
	A14	TK4888	S	1	22/06/2014 11:57	5.039333 E 42.119199 N	GPS			419 82°	lost	n

For each bird, treatment (Tr), track code (Track), ring number (Ring), breeding Colony (S, La Scola; B, Punta Brigantina), number of days between the incubation shift and the bird capture (SD), date and time of release, Longitude and Latitude (Location) of the release point are reported. In the Tag column the kind of logger used is specified. The following columns report details of mean vector calculations done for the section of the tracks up to 60 km from the release point: n, number of angles used to calculate the mean vectors; mv, mean vector length and direction; home distance (km) and direction (°) from the release point. Homing: date of bird homing or recapture (h or r, respectively); lost indicates that the bird never returned to their nest during the breeding period. Track analysis: availability of tracking data for analysis is indicated as follows; c: complete track from the release point to home; i: incomplete track; lo: low number of fixes; n: no track obtained. The parameters included in the analysis are indicated by the numbers as follows: [1]homing performances; [2]mean vector length in the first 60 km; [3]Efficiency Index relative to the section of track from the release point up to 10 km from home; [4]preference of the birds in travelling near the coast; [5]decision point.

Table S2

Code	Ring	Release	Homing	GPS off	Flight	MD	Length	Duration	Track
C2	TJ3757	21/06/2012	22/06/2012	24/06/2012	23/06/2012	31.2	121.7	18^{h} 32’	
C3	TJ9179	21/06/2012	22/06/2012	26/06/2012	24/06/2012	7.7	48.9	19^{h} 00’	
					25/06/2012	17.5	108.1	18^{h} 09’	
					26/06/2012	18.2	129.1	18^{h} 44’	
C8	TK4887	22/06/2014	24/06/2014	27/06/2014	25/06/2014	30.4	123.2	22^{h} 14’	
					26/06/2014	32.5	160.4	19^{h} 06’	
					27/06/2014	22.8	83.0	20^{h} 40’	
M6	TH0410	26/06/2012	27/06/2012	29/06/2012	28/06/2012	12.9	94.8	17^{h} 23’	
A1	TH0433	21/06/2012	27/06/2012	02/07/2012	27/06/2012	45.4	169.1	20^{h} 12’	
					29/06/2012	98.5	855.0	91^{h} 48’	
A9	TH0438	22/06/2014	25/06/2014	05/07/2014	26/06/2014	31.8	190.4	17^{h} 06’	
					27/06/2014	28.5	100.1	17^{h} 25’	
					28/06/2014	26.8	110.7	18^{h} 20’	
					29/06/2014	75.7	287.6	19^{h} 00’	
					30/06/2014	42.0	180.3	21^{h} 29’	
					01/07/2014	47.0	154.9	18^{h} 32’	
					02/07/2014	33.5	141.0	17^{h} 45’	
					03/07/2014	46.8	193.0	18^{h} 33’	
					04/07/2014	49.4	282.3	18^{h} 13’	
A12	TA2247	22/06/2014	05/07/2014	07/07/2014	06/07/2014	25.0	77.9	20^{h} 38’	

For each bird, the individual code (Code), ring number (Ring), date of release (Release), date of homing after displacement (Homing), date of the GPS power off (GPS off), date of start of each spontaneous flight (Flight). For each spontaneous flight the maximal distance (km) reached from the colony (MD), the length (km) of the flight path (Length), and the duration of the journey (Duration) are reported. For each flight the identification colour of the track reported in Figure S1 is shown (Track).
